# Association of GSTM1 and GSTT1 Copy Number Variation with Chromosomal Aberrations in Nuclear Power Plant Workers Exposed to Occupational Ionizing Radiation

**DOI:** 10.3390/toxics13020073

**Published:** 2025-01-22

**Authors:** Joong won Lee, Younghyun Lee, Yang Jee Kim

**Affiliations:** 1 Division of Population Health Research, Department of Precision Medicine, Korea National Institute of Health, Korea Disease Control and Prevention Agency, Cheongju 28159, Republic of Korea; won3@korea.kr; 2 Department of Biomedical Laboratory Science, College of Medical Sciences, Soonchunghyang University, 22, Soonchunhyango-ro, Asan-si 31538, Republic of Korea; ylee0123@sch.ac.kr; 3 College of General Education, Chung-Ang University, 84 Heukseok-ro, Dongjak-gu, Seoul 16974, Republic of Korea

**Keywords:** ionizing radiation, chromosome aberration, copy number variation, glutathione S-transferase mu-1 (GSTM1), glutathione S-transferase theta-1 (GSTT1)

## Abstract

Exposure to low-dose ionizing radiation in occupational settings raises concerns about chromosomal aberrations (CAs) and their potential impact on genomic stability. Copy number variations (CNVs), structural genomic changes, influence susceptibility to environmental stressors and radiation-induced damage. This study analyzed CAs in 180 nuclear power plant workers exposed to occupational radiation and 45 controls, stratified by GSTM1 and GSTT1 CNVs. Workers exhibited significantly higher frequencies of chromatid-type and chromosome-type aberrations, of 5.47 and 3.01 per 500 cells, respectively, compared to 3.57 and 0.64 in controls (*p* < 0.001 for both). In the relatively high-exposure group, chromatid-type aberrations decreased with increasing GSTM1 and GSTT1 copy numbers. For GSTM1, individuals with zero copies showed 6.37 ± 3.47 aberrations per 500 cells, compared to 5.02 ± 3.05 for one copy and 4.67 ± 2.40 for two or more copies (*p* = 0.06). A similar trend was observed for GSTT1, with 6.00 ± 3.29 aberrations per 500 cells for zero copies, 5.38 ± 2.79 for one copy, and 4.11 ± 4.26 for two or more copies (*p* = 0.05). Poisson regression analysis further supported these findings after adjusting for potential confounders such as age, smoking status, and alcohol intake. Workers with null genotypes exhibited a 1.36-fold increase in chromatid-type aberrations compared to those with higher copy numbers under relatively high-exposure conditions, suggesting a synergy effect between GSTM1 and GSTT1 null genotypes in modulating radiation-induced aberrations. These findings underscore the role of genetic susceptibility, particularly involving GSTM1 and GSTT1 CNVs, in modulating radiation-induced chromosomal damage. The observed gene–environment interaction in the relatively high-exposure group suggests that pre-existing CNVs contribute to chromosomal instability under radiation exposure.

## 1. Introduction

Ionizing radiation (IR) is widely utilized across diverse industries, notably in healthcare, research, and nuclear power generation. As reliance on IR continues to grow, the risks associated with occupational exposure, even at low doses, demand increasing attention. Despite strict regulations by organizations such as the International Commission on Radiological Protection (ICRP), prolonged low-dose IR exposure poses significant health concerns, particularly in terms of its genotoxic effects [1,2]. Radiation-induced chromosomal aberrations (CAs) are well-studied markers of genomic instability and are critical in understanding the biological effects of radiation. These aberrations reflect cumulative damage over time and contribute to long-term health complications [3].

In occupational health research, identifying biomarkers for individual radiosensitivity has become crucial for risk assessment and worker protection strategies [4]. Chromosomal aberrations detected in peripheral blood lymphocytes (PBLs) have been extensively utilized as biomarkers to assess genotoxic effects caused by ionizing radiation [5,6] and to explore the potential role of high CA frequencies as an early marker of cancer risk [7,8,9].

IR primarily causes damage through two mechanisms: direct DNA ionization and the generation of reactive oxygen species (ROS), both of which lead to significant DNA damage [10,11]. ROS-driven oxidative stress exacerbates DNA damage and promotes mutations, increasing cancer risk [12]. In this context, the glutathione S-transferase (GST) enzyme family plays a critical role in neutralizing ROS and detoxifying harmful metabolites [13]. Among these enzymes, GSTM1 and GSTT1 are particularly notable for mitigating oxidative damage. Polymorphisms in the genes encoding these enzymes, particularly deletion variants, reduce or eliminate enzyme activity, increasing susceptibility to genotoxic effects. These genetic deletions have also been associated with a heightened risk of cancer and other diseases, highlighting their potential as biomarkers for IR-induced damage [14,15].

While numerous studies have explored the role of GST polymorphisms in cancer and other oxidative stress-related conditions, the association between GSTM1 and GSTT1 deletions and susceptibility to IR-induced chromosomal aberrations in occupational settings remains underexplored. Previous studies often faced limitations such as small sample sizes or oversimplified genotyping approaches, which may have underestimated the true associations between GSTM1 and GSTT1 copy numbers and IR-induced DNA damage [16,17]. More precise determination of gene copy numbers now allows for a detailed investigation of their potential contribution to radiosensitivity in exposed populations [18].

The primary aim of this study is to assess the association between GSTM1 and GSTT1 copy number variations and the frequency of chromosomal aberrations in nuclear power plant workers exposed to low-dose IR. By examining these genetic factors in relation to chromosomal aberration data, this research seeks to provide insights into the role of GST polymorphisms in modulating the genotoxic effects of radiation exposure.

## 2. Materials and Methods

### 2.1. Ethics Statement

The study protocol was approved by the Research Ethics Review Board of Seoul National University. All participants provided written informed consent before inclusion in the study.

### 2.2. Study Population

This study builds on previous research investigating chromosomal damage in nuclear power plant workers exposed to low-dose ionizing radiation [19]. The study included 108 male workers occupationally exposed to low doses of ionizing radiation (IR) at four nuclear power plants in South Korea (Kori, Wolsong, Yonggwang, and Ulchin). The control group consisted of 45 male office workers employed at the same nuclear power plants. These individuals were not occupationally exposed to radiation, as their job roles did not involve direct contact with radiation sources. Blood samples from both groups were collected during the same period. Occupational radiation doses were determined using official personal dosimetry records obtained from the Korean National Dose Registry, managed by the Korea Radio-Isotope Association (KRIA).

### 2.3. DNA Isolation and Copy Number Assays

Genomic DNA was isolated from 2 mL of peripheral whole blood using the Wizard™ DNA Purification Kit (Promega, Madison, WI, USA). The protocol involved cell lysis, protein precipitation, and DNA precipitation with isopropanol. After ethanol washing, the DNA was resuspended in TE buffer and stored at −20 °C until analysis. DNA concentration and purity were checked using a NanoDrop spectrophotometer, ensuring OD 260/280 ratios between 1.8 and 2.0. The copy numbers of GSTM1 and GSTT1 were determined using quantitative real-time PCR (qPCR) with TaqMan^®^ Copy Number Assays (Applied Biosystems, USA). The Hs02575461_cn assay was used for GSTM1, and Hs00010004_cn for GSTT1, with the RNase P gene as a reference to normalize copy number estimates. Each qPCR reaction (20 μL) contained TaqMan^®^ Universal PCR Master Mix, the respective TaqMan Copy Number Assay, and a DNA sample diluted to 5 ng/μL. The qPCR reactions were run in triplicate on a 7300 Real-Time PCR System. The cycling conditions included an initial denaturation at 95 °C for 10 min, followed by 40 cycles of 95 °C for 15 s and 60 °C for 1 min.

The comparative ΔΔCt method was applied to calculate the relative copy number. Ct values from GSTM1 and GSTT1 were normalized to those of the reference gene (RNase P) in each sample. A calibrator sample with known two-copy status was used for accuracy, and the relative quantity (2^ΔΔCt) was multiplied by two to estimate copy numbers in the diploid genome. No-template controls were included in each plate to control for contamination. For quality control, any samples with standard deviations greater than 0.5 in Ct values among triplicates were flagged for re-analysis. Random samples were also repeated, yielding 100% concordance with the initial results.

### 2.4. Chromosome Aberration (CA) Assay

Peripheral blood samples were collected from each participant in heparinized tubes. Samples were processed immediately, and 1 mL of blood was seeded in 9 mL of RPMI 1640 culture medium supplemented with fetal bovine serum (10%), antibiotics, and phytohemagglutinin to stimulate lymphocyte growth. The cultures were incubated at 37 °C in a humidified atmosphere containing 5% CO_2_ for 48 h. Colcemid (0.1 μg/mL) was added 3 h before harvesting to arrest cells in metaphase. Chromosome preparations were performed according to the standard procedure [20]. Slides were coded blindly, and 500 metaphases for each subject were scored randomly. The aberration types, including chromatid type and chromosome type, were counted separately.

Fluorescence plus Giemsa (FPG) staining for sister chromatids [21] was performed to differentiate between first and second cell divisions. Chromosomal aberrations were analyzed exclusively in cells from the first division, confirmed by BrdU incorporation, which showed that 87–95% (average 90.5%) of observed cells were in the first division.

### 2.5. Statistical Analysis

Data were analyzed using SAS 9.1 software (SAS Institute Inc., Cary, NC, USA). Differences in the frequency of chromosome aberrations between nuclear power plant workers and controls were analyzed by the Mann–Whitney U-test. Kruskal–Wallis Tests and Mann–Whitney U-tests were employed to compare GST copy numbers and chromosomal aberrations, as well as to examine GST copy number distributions between radiation-exposed workers and controls. Poisson regression models were subsequently used to estimate frequency ratios (FRs) of aberrations, adjusting for age, smoking, alcohol consumption, and radiation dose, with differences considered statistically significant at *p* < 0.05.

## 3. Results

### 3.1. General Characteristics of Study Population

The general characteristics of the study population are summarized in Table 1. A total of 180 radiation-exposed workers and 45 control subjects were included in the study. The mean age of the radiation-exposed workers was 47.5 ± 5.9 years, while the control subjects had a mean age of 40.9 ± 10.0 years. There were no significant differences in smoking status between the two groups. However, a significant difference was found in alcohol consumption, with 81.7% of radiation-exposed workers reporting alcohol use compared to 66.7% of the controls (*p* = 0.01). The cumulative radiation dose for the exposed workers ranged from 12.7 to 400.3 mSv, with a mean of 157.3 ± 85.2 mSv.

### 3.2. Chromosomal Aberration Frequency

Radiation-exposed workers exhibited significantly higher chromosomal aberration (CA) frequencies compared to controls (Table 2). The mean frequency of chromatid-type aberrations was 5.47 per 500 metaphase cells in the exposed workers, significantly higher than 3.57 in the controls (*p* < 0.001). Similarly, the mean frequency of chromosome-type aberrations was 3.01 per 500 cells in the exposed workers, compared to 0.64 in the controls (*p* < 0.001). These significant differences remained even after adjusting for confounding factors such as age, smoking status, and alcohol consumption using Poisson regression analysis.

### 3.3. GSTM1 and GSTT1 Copy Number Distributions

The distributions of GSTM1 and GSTT1 copy numbers in radiation-exposed workers and controls are shown in Table 3. There were no statistically significant differences between the two groups in terms of the copy number distribution for both genes. In the study population, the frequency of GSTM1 null genotypes (zero copies) was 50.67%, and for GSTT1, it was 49.78%, which is similar to previously reported values for East Asian populations (GSTM1 null: 52%, GSTT1 null: 48%) [22]. The frequencies of individuals with two copies of GSTM1 and GSTT1 were 8.89% and 9.78%, respectively.

### 3.4. Association Between GST Copy Number and Chromosomal Aberrations

As shown in Table 4, no significant correlation was found between GSTM1 and GSTT1 copy numbers and chromosomal aberration frequencies across groups with zero, one, or two or more copies. Individuals with two copies of GSTM1 or GSTT1 only exhibited a slightly lower mean frequency of chromosome aberrations compared to those with zero copies or one copy, but there was no statistical significance.

For further analyses, exposed workers were categorized into two groups based on their recent 1.5-year radiation exposure levels: the relatively low-exposure group (≤7.5 mSv, *n* = 89) and the relatively high-exposure group (≥7.5 mSv, *n* = 91).

**Figure 1:**
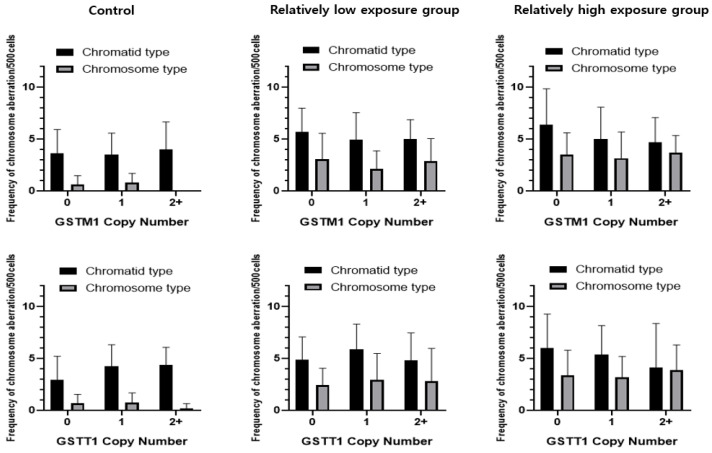
Frequency of chromatid-type and chromosome-type CAs according to GSTM1 and GSTT1 copy numbers in controls and relatively low- and high-exposure worker groups.

Figure 1 illustrates the relationships between GSTM1 and GSTT1 copy numbers and chromosomal aberration frequencies across different radiation exposure levels. In the high-exposure group, higher copy numbers of GSTM1 and GSTT1 were associated with lower frequencies of chromatid-type aberrations. For GSTM1, individuals in the relatively high-exposure group with zero copies exhibited a higher frequency, of 6.37 ± 3.47 per 500 cells, compared to 5.02 ± 3.05 for one copy and 4.67 ± 2.40 for two copies (*p* = 0.06). Similarly, for GSTT1, frequencies in the relatively high-exposure group decreased from 6.00 ± 3.29 for zero copies to 5.38 ± 2.79 and 4.11 ± 4.26 for one and two copies, respectively (*p* = 0.05).

Multivariate analysis further confirmed an inverse relationship between GSTM1 and GSTT1 copy numbers and the frequency of chromatid-type aberrations in the high-exposure group, even after adjusting for confounders such as smoking, age, alcohol intake, and recent radiation exposure dose (Table 5). For GSTM1, individuals with one or more copies exhibited lower chromatid-type aberration frequencies compared to those with zero copies (e.g., FR = 0.77, 95% CI = 0.64–0.93 for one copy; FR = 0.71, 95% CI = 0.51–0.98 for two or more copies). A similar trend was observed for GSTT1, where frequencies decreased progressively from zero copies (reference) to one copy (FR = 0.93, 95% CI = 0.77–1.13) and two or more copies (FR = 0.70, 95% CI = 0.49–0.99).

**Figure 2:**
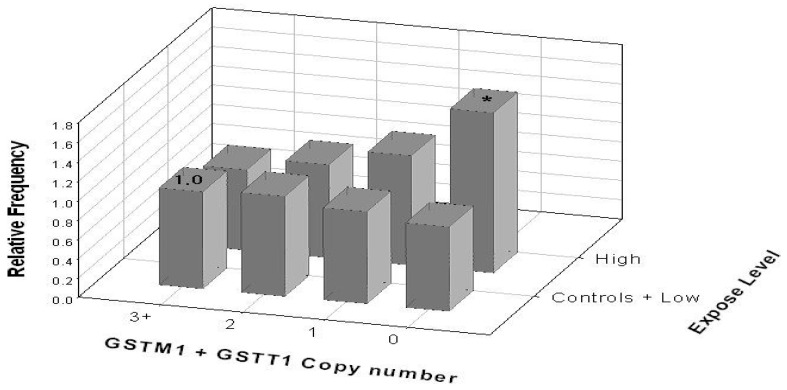
Interaction between GST copy number and radiation exposure in the occurrence of chromatid-type aberrations; *, *p* < 0.05 compared to the reference (for which the relative frequency ratio is 1.0).

As shown in Figure 2, individuals carrying double-null genotypes had a 1.36-fold increase (95% CI: 1.03–1.81) in chromatid-type aberrations compared to those with the highest copy number (reference group) in the high-exposure group. This highlights the combination of GSTM1 and GSTT1 copy numbers’ interaction with radiation exposure levels.

## 4. Discussion

This study highlights the role of GSTM1 and GSTT1 copy number variations in influencing susceptibility to radiation-induced chromosomal aberrations (CAs) among nuclear power plant workers.

The CA frequency was significantly higher in radiation-exposed workers compared to controls, consistent with earlier findings [6,23,24]. Chromatid-type aberrations were particularly dominant in our study, as also demonstrated by Shatha [25], which highlighted the prominence of chromatid-type damage in in vitro low-dose conditions. This difference may be attributed to radiation-induced reactive oxygen species (ROS), which drive genomic instability and DNA damage. At lower radiation doses, ROS play a critical role in promoting chromatid-type aberrations, likely through the induction of oxidative stress and subsequent genomic instability [26,27]. Under prolonged oxidative stress, ROS-induced SSBs may persist and convert into DSBs during replication, contributing to genomic instability [27,28]. As described by Kryston et al. [29], oxidative DNA damage can lead to clustered lesions, which are challenging to repair and may be associated with chromatid-type aberrations.

GST copy number variations did not show a significant correlation with chromosomal aberrations in our study (Table 4). A similar lack of association was reported by Gulati et al. [17], who found no direct link between GST polymorphisms and DNA damage in individuals exposed to mobile tower radiation. When we considered radiation exposure levels, an inverse association between GSTM1 and GSTT1 copy numbers and chromosomal aberrations became evident, particularly in occupational settings with relatively high radiation exposure. Individuals with null genotypes showed higher chromosomal damage, suggesting that genetic predisposition plays a crucial role in determining radiosensitivity under heightened environmental stress [10,11,30]. Furthermore, individuals with double-null genotypes exhibited the highest chromosomal aberration frequencies, indicating a combined effect of GSTM1 and GSTT1 deficiencies in amplifying radiation-induced oxidative stress. This combination highlights a synergy effect in modulating radiation-induced aberrations, consistent with previous studies reporting increased chromosomal damage in radiation workers and individuals exposed to various genotoxic agents [31,32]. This combined effect suggests a gene–environment interaction, where genetic susceptibility becomes more pronounced under environmental stressors such as radiation exposure, as predicted by Ottman’s model [33]. The synergistic effect of GSTM1 and GSTT1 null genotypes on chromosomal aberrations highlights the necessity of incorporating gene–environment interactions into occupational radiation risk assessments.

The role of GSTM1 and GSTT1 enzymes in detoxifying ROS provides a possible explanation for these observations. When these enzymes are absent due to null genotypes, ROS can accumulate, increasing oxidative stress and resulting in chromatid-type aberrations [7,25,34]. This gene dosage effect, where increased copy numbers enhance enzymatic activity, was evident in our findings. Given that oxidative stress plays a key role in radiation-induced genomic instability, previous studies have also reported that GSTM1 and GSTT1 null genotypes are linked to heightened oxidative stress in radiation-exposed workers [35], further reinforcing their role in modulating radiosensitivity. Individuals with one or more copies of GSTM1 and GSTT1 showed lower chromosomal aberration frequencies compared to those with null genotypes, demonstrating the protective role of these enzymes against oxidative stress [30,36]. Similarly, Glushkov et al. [31] demonstrated that GSTM1 and GSTT1 null genotypes enhance chromosomal damage under environmental stressors, such as smoking and occupational chemical exposure. In addition, Zeng et al. [37] showed that GSTM1 null and GSTT1 null genotypes increase susceptibility to respiratory inflammation caused by particulate matter in air pollution. Together, these findings underscore the role of GST polymorphisms in modulating the biological impact of environmental stressors.

Genetic analyses in occupational settings can provide valuable insights into individual susceptibility, complementing physical dosimetry by integrating biological markers such as chromosomal aberrations to improve radiation exposure assessment. However, further research with larger sample sizes and the inclusion of diverse genetic factors is essential to validate these findings and better understand their role in modulating genomic instability under varying levels of radiation exposure.

## 5. Conclusions

This study highlights the significant role of GSTM1 and GSTT1 copy number variations in determining susceptibility to chromosomal aberrations in nuclear power plant workers exposed to low-dose ionizing radiation. Null genotypes for these genes were associated with higher frequencies of chromatid-type aberrations, indicating increased vulnerability to radiation-induced damage.

## Figures and Tables

**Table 1:** General characteristics of study population.

Variables	No. of Subjects (%)		*p*-Value
	Controls	Workers	
Number	45	180	
Age (mean ± SD, years)	40.9 ± 10.0	47.5 ± 5.9	0.01 ^a^
≤50	36 (80)	117 (65)	0.05 ^b^
>50	9 (20)	63 (35)	
Smoking status (mean ± SD, pack-years)	14.6 ± 13.5	12.6 ± 11.3	0.52 ^a^
Current smoking	20 (44.4)	69 (38.3)	0.45 ^b^
Never smoking	25 (55.6)	33 (18.3)	
Alcohol intake			
Yes	30 (66.7)	147 (81.7)	0.03 ^b^
No	15 (33.3)	33 (18.3)	
Duration of employment (mean ± SD, years)	-	20.1 ± 6.2	
≤20	-	103 (45.8)	
>20	-	122 (54.2)	
Dosimetry radiation dose			
Recent 1.5-year (mean ± SD, mSv)	-	8.2 ± 7.1	
Cumulative dose (mean ± SD, mSv)	-	157.3 ± 85.2	

SD, Standard deviation. ^a^ Determined by Mann–Whitney U-test. ^b^ Determined by *x*^2^ test.

**Table 2:** Frequencies of chromosomal aberrations in nuclear power plant workers and controls.

Types of Chromosome Aberration	Controls			Radiation Exposed Workers			
	Mean/ 500 Cells	SD	Range	Mean/ 500 Cells	SD	Range	*p*-Value *
Number	45			180			
Chromatid-type deletion	3.53	2.21	0- 10	5.41	2.76	0–17	<0.0001
Chromatid-type exchange	0.04	0.21	0–1	0.06	0.23	0–1	0.7697
Chromosome-type deletion	0.51	0.82	0–3	2.08	1.98	0–14	<0.0001
Chromosome-exchange	0.13	0.40	0–2	0.93	1.02	0–6	<0.0001
Total aberration	4.09	2.51	1–12	8.39	3.53	1–19	<0.0001

SD, standard deviation. * determined by Mann–Whitney U-test.

**Table 3:** Distribution of GSTM1 and GSTT1 copy number in radiation-exposed workers and controls.

	Copy Number	Controls, N (%)	Exposed, N (%)	*p*-Value *
No. of Subjects		45	180	
GSTM1	0 ^a^	25 (55.56)	89 (49.44)	0.65
	1 ^b^	17 (37.38)	72 (40.00)	
	2 ^c^	3 (6.67)	17 (9.44)	
	3+	0 (0.00)	2 (1.11)	
	0	25 (55.56)	89 (49.44)	0.47
	1+	20 (44.44)	91 (50.56)	
	0 or 1	42 (93.33)	161 (89.44)	0.43
	2	3 (6.67)	19 (10.56)	
GSTT1	0 ^a^	24 (53.33)	88 (48.89)	0.75
	1 ^b^	16 (35.56)	75 (41.67)	
	2 ^c^	5 (11.11)	17 (9.44)	
	0	24 (53.33)	88 (48.89)	0.59
	1+	21 (49.67)	92 (51.11)	
	0 or 1	40 (88.89)	163 (90.56)	0.74
	2+	5 (11.11)	17 (9.44)	

* The difference in copy numbers between radiation-exposed workers and controls, *x*^2^ test. ^a^, (0/0) Homozygous deletion (null genotype). ^b^, (0/1) Hemizygous deletion. ^c^, (1/1) Wild-type (positive genotype, two functional genes).

**Table 4:** Frequency of chromosome aberrations (CAs) in radiation-exposed workers and controls according to GSTM1 and GSTT1 copy numbers.

	Copy Number	Controls			Exposure Group		
		N	CA ± SD	*p*-Value	N	CA ± SD	*p*-Value
GSTM1	0 ^i^	25	4.04 ± 2.76	0.75 ^a^	89	8.97 ± 3.50	0.04 ^a^
	1 ^ii^	17	4.29 ± 2.28		72	7.75 ± 3.62	
	2+ ^iii^	3	3.33 ± 2.08		19	8.16 ± 3.04	
						
	0 or 1	42	4.41 ± 2.55	0.65 ^b^	161	8.42 ± 3.59	0.95 ^b^
	2+	3	3.33 ± 2.08		19	8.16 ± 3.04	
GSTT1	0 ^i^	24	3.50 ± 2.73	0.07 ^a^	88	8.22 ± 3.35	0.43 ^a^
	1 ^ii^	16	4.81 ± 2.20		75	8.75 ± 3.46	
	2 ^iii^	5	4.60 ± 1.95		17	7.76 ± 4.70	
						
	0 or 1	40	4.03 ± 2.59	0.44 ^b^	163	8.46 ± 3.40	0.38 ^b^
	2+	5	4.60 ± 1.95		17	7.76 ± 4.70	

CA, chromosome aberration frequency per 500 cells. SD, standard deviation. ^a^, Tested by Kruskal–Wallis Test. ^b^, Tested by Mann–Whitney U-test; ^i^ (0/0), homozygous deletion (null genotype); ^ii^ (0/1), hemizygous deletion. ^iii^ (1/1), Wild-type (positive genotype, two functional genes).

**Table 5:** Poisson regression analysis of chromosome aberrations (CAs): effects of GSTM1 and GSTT1 copy number and radiation exposure in the relatively highly exposed group.

	Copy Number	Total Chromosome Aberration			Chromosome-Type Aberration			Chromatid-Type Aberration		
		FR ^a^	95% CI	*p*-Value	FR	95% CI	*p*-Value	FR	95% CI	*p*-Value
GSTM1	0	1.00	reference		1.00	reference		1.00	reference	
	1	0.87	0.75 1.01	0.06	0.92	0.72 1.17	0.48	0.77	0.64 0.93	0.01
	2+	0.86	0.66 1.09	0.23	1.02	0.68 1.47	0.93	0.71	0.51 0.98	0.04
GSTT1	0	1.00	reference		1.00	reference		1.00	reference	
	1	0.97	0.83 1.12	0.65	0.97	0.75 1.24	0.79	0.93	0.77 1.13	0.48
	2	0.90	0.69 1.15	0.41	1.17	0.79 1.69	0.42	0.70	0.49 0.99	0.05

Relatively high-exposure group. (Recent 1–5 yr dose, ≥7.5 mSv; *n* = 91/180). ^a^, Frequency ratio adjusted for age, smoking status, and alcohol intake.

## Data Availability

The data presented in this study are available on request from the corresponding author.
